# Unruptured tubal ectopic pregnancy concurrent with trophoblastic tissue spread to the rectum: a case report

**DOI:** 10.1097/MS9.0000000000002141

**Published:** 2024-05-15

**Authors:** Kareem Zabad, Massa Tarakji, Raghad Arafat, Yeser Al-Douni, Merry Nakhleh, Bashar Kurdi

**Affiliations:** a Faculty of Medicine, Damascus University; b University Hospital of Obstetrics and Gynecology; c Syrian Private University, Damascus, Syria

**Keywords:** case report, ectopic pregnancy, obstetrics, persistent trophoblastic tissue trophoblastic implant

## Abstract

**Introduction and importance:**

Ectopic pregnancy is a term used to describe pregnancies outside of the uterus. If ruptured, persistent trophoblastic tissue can be present at the site of an ectopic pregnancy, which is an unusual complication. In rare cases, the patients may present with primary implants.

**Case presentation:**

A 23-year-old woman who was pregnant for the first time complained of abdominal pain and was diagnosed with a 10-week tubal ectopic pregnancy. During laparotomy, trophoblastic implants were discovered in the rectum; however, the pregnancy did not rupture.

**Clinical discussion:**

The presented case report highlights a rare and late diagnosis of ectopic pregnancy in a 23-year-old primigravida woman with no known risk factors. The patient experienced sudden abdominal pain in the tenth week of gestation, leading to the surgical approach of salpingectomy. Notably, trophoblastic tissue was found in the rectum, indicating local invasion. It was then treated with methotrexate therapy. However, the low-resource setting in Syria limited the use of laparoscopy and resulted in the use of laparotomy.

**Conclusion:**

This case emphasizes the importance of considering trophoblastic implantation in the management plan for ectopic pregnancies, even in cases in which the pregnancy has not ruptured. It is crucial to evaluate all possible complications of this disease to ensure proper treatment and care of the patient.

## Introduction

Highlights

- Ectopic pregnancy is a disease that requires the utmost attention of caregivers.
- If a ruptured ectopic pregnancy is not treated properly, residual products of conception can persist outside the uterus and may cause high serum human chorionic gonadotropin (hCG) readings and even dangerous complications.
- Exceptionally, trophoblastic tissue implants are discovered as a primary entity that should be taken care of promptly.

Ectopic pregnancy refers to a pregnancy that takes place outside of the uterus^1^. Particularly, in the fallopian tubes, which account for the majority of ectopic pregnancies, while the remaining cases occur in the ovary, cervix, or abdominal cavity^2^.

Although ectopic pregnancy accounts for ~1–2% of first-trimester pregnancies, it is considered a significant entity as it causes nearly 6% of pregnancy-related deaths due to complications^3^. Furthermore, typical symptoms include abdominal pain, amenorrhea, and sometimes vaginal bleeding. However, in the first gestational weeks, half the patients may be asymptomatic or present atypically^3,4^. Transvaginal ultrasonography is the gold standard for diagnosing ectopic pregnancy. It is usually used in conjunction with quantitative serum human chorionic gonadotropin (hCG) testing^5^.

Ectopic pregnancy poses a significant risk of bleeding and tubal rupture, which can lead to maternal mortality if not addressed promptly. Additionally, there is a possibility of leaving behind fetal tissue. It is crucial to closely monitor the medial portion of the tube, as it is the most likely site for trophoblastic tissue to persist, causing elevated serum A-hCG levels during follow-up^6^.

Persistent trophoblast tissue is usually present at the surgical site of ectopic pregnancy as an uncommon complication or as primary implants in unique cases^7^.

In this report, we present an exceptional case of a primigravida woman with no prior surgical history, who was diagnosed with an unruptured tubal ectopic pregnancy, along with displaying trophoblastic tissue implants in the rectum during surgery. The objective of this case study was to highlight the rarity of this condition and raise awareness among healthcare professionals.

This case report is in accordance with the SCARE 2023 criteria^8^.

### Case presentation

A 23-year-old primigravida visited the University Hospital of Obstetrics complaining of abdominal discomfort. Her last menstrual period was 10 weeks, and she had no prior medical or surgical history, allergies, or medication use. Family history was also unremarkable. Additionally, she did not receive any assisted reproductive treatment and showed no signs of sexually transmitted infection.

During her visit, she reported experiencing abdominal pain that began 24 h prior without any preceding injuries. The pain was constant, had not spread to other areas, and was not accompanied by bleeding or discharge. There were no other symptoms or fevers.

**Figure 1:**
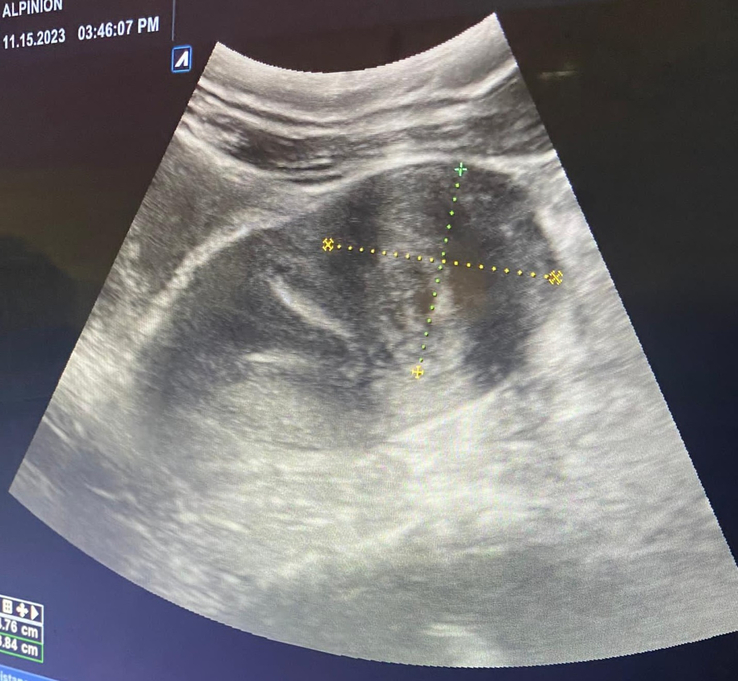
Pelvic ultrasound showing subserosal fibroid in the uterus.

**Figure 2:**
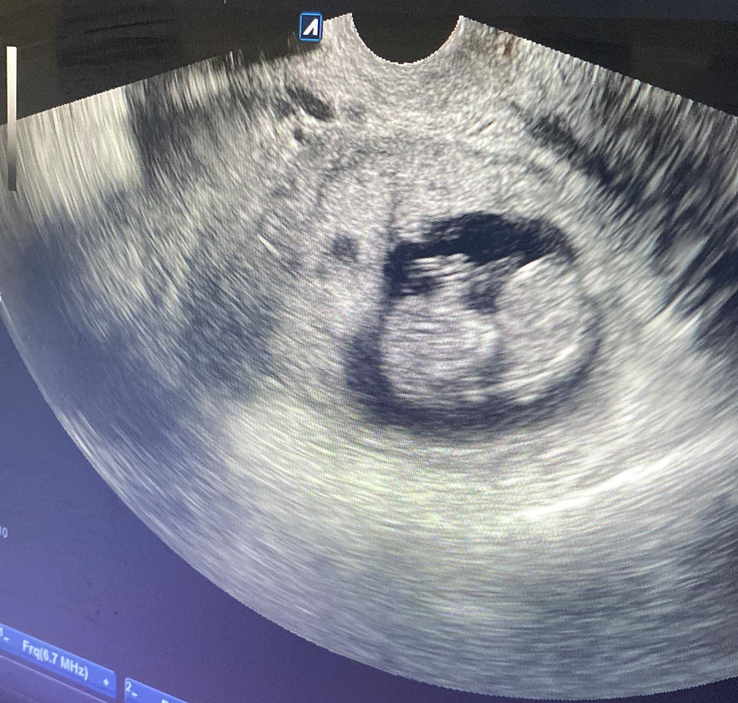
Trans-vaginal ultrasound showing uterine decidual reaction no gestational sac within the uterine cavity, and a gestational sac at the left fallopian tube pedunculated posterolateral and surrounded by myosalpinx.

Upon clinical examination, the patient’s blood pressure was 110/70 mmHg with a heart rate of 84 beats per min. The cervix was closed, not dilated, and there was no bleeding. A bedside pelvic ultrasound revealed a heterogeneous echogenic lesion measuring 3.3×4.7 cm with calcifications, indicating a subserosal fibroid [Figure 1]. Transvaginal ultrasound showed an empty uterine cavity with a decidua reaction measuring 1.6 mm and an extrauterine gestational sac measuring 7×5.2 cm in the left fallopian tube. The Crown Rump length (CRL) measured 1.9 cm, indicating a gestational age of 10 weeks with cardiac activity [Figure 2]. Her BhCG level increased to 45 000 IU/L. No free fluids were detected in the abdomen, and tubal ectopic pregnancy was diagnosed.

Her hemoglobin count measured 9.5 g/l, her hematocrit 27.3 g/l, PT 11s, her PTT 28s compared to the normal range of 9.4–12.5s and 25–37s respectively, and her total white cell count was 17.2 g/l. Blood grouping and cross-matching of the four blood units were immediately performed.

**Figure 3:**
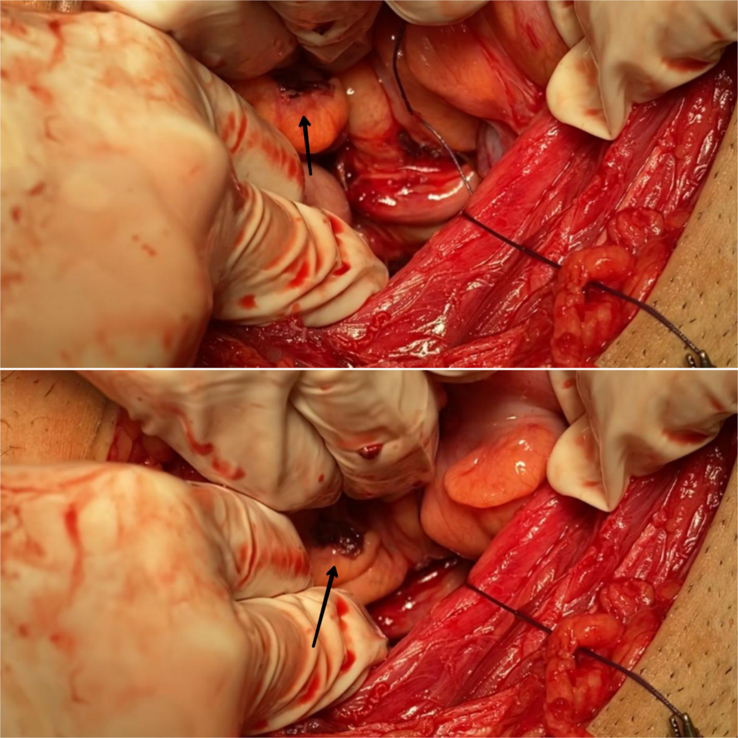
Trophoblastic implant on the rectum.

Due to the presence of an extrauterine pregnancy, laparotomy was performed under spinal anesthesia through a Pfannenstiel incision. An unruptured ectopic pregnancy was detected in the left fallopian tube, which was surgically resected along the tube. Additionally, a subserosal fibroid was found in the uterus and was resected. Non-bleeding implantation was observed in the rectum. Gross features, similarity with the implantation found on the tube, and clinical exclusion of other pathologies led to the diagnosis of trophoblastic implantation [Figure 3]. No histopathological examination was made as the implantations bled with a fine touch, and the surgeons decided against a biopsy. The abdominal cavity was irrigated, and a ZZ drain was placed in the Douglas pouch. The layers were sutured, and a 2-0 nylon suture was used to suture the skin cosmetically. The patient received 50 mg of intramuscular methotrexate and recovered well, leading to discharge from the hospital. Follow-up appointments were scheduled to monitor hCG levels. The patient missed the weekly appointments but returned after a month, and hCG levels were below 5 IU/l while also feeling well after the surgery.

## Discussion

An ectopic pregnancy is when a pregnancy occurs outside of the uterus^1^. In 97% of cases, it arises in the fallopian tubes, particularly in the ampulla (55%), isthmus (25%), and fimbria (17%). Whereas in a small percentage (3%) of patients, it occurs in the abdominal cavity, ovary, or cervix^2^.

Patients with ectopic pregnancy commonly experience symptoms such as abdominal pain, absence of menstrual periods, and occasional vaginal bleeding. However, it’s important to note that some patients may have atypical symptoms or remain symptom-free, until it is detected in a routine visit, or after becoming symptomatic, due to rupture around the seventh week of gestation (7.2 ± 2.2 weeks). At this stage, the patient may present with severe abdominal pain, which can progress to shock and even death if not promptly addressed. Thus, a late diagnosis has significant implications as it increases the risk of rupture and narrows the options of treatment down to surgery^3,9,10^.

Our patient had an ectopic pregnancy in the fallopian tube, which went unnoticed until the tenth week of gestation when she experienced sudden abdominal pain. This is considered a late diagnosis, and some authors believe that any tubal pregnancy beyond the tenth week is advanced^11^.

Previous ectopic pregnancy, tubal surgery, documented tubal pathology, exposure to diethylstilbestrol, a history of genital infections such as pelvic inflammatory disease (PID), chlamydia, gonorrhea, infertility, having multiple sexual partners, smoking, and advanced maternal age are all associated with an elevated risk of ectopic pregnancy^12,13^.

In our case, none of the risk factors were present. A 23-year-old primigravida woman had no history of surgery, previous pregnancies, smoking, STDs, or assisted reproduction procedures.

Surgery is necessary when a patient with ectopic pregnancy experiences hemodynamic instability, ongoing rupture of the ectopic mass, intraperitoneal bleeding, or suspicion of heterotopic pregnancy. Additionally, surgery is required for patients with absolute contraindications to medical management. Salpingectomy is preferred to salpingotomy. However, salpingotomy may also be used in patients with a history of infertility^1^.

In hemodynamically stable women with an unruptured tubal ectopic pregnancy and no signs of active bleeding, a fixed multiple-dose intramuscular regimen of systemic methotrexate may be a suitable treatment option for those presenting with serum hCG concentrations below 3000 IU/l^10^.

It is worth mentioning that the choice of therapy can affect the chance of recurrence^12^.

The surgical approach was chosen for this patient. A salpingectomy was performed followed by methotrexate therapy. The patient showed no recurrence after a month.

Persistent trophoblastic tissue is a rare complication that can occur because of incomplete treatment of ectopic pregnancy, often due to conservative surgical approaches. This tissue is typically found in the medial portion of the tube or at the surgical site and can lead to elevated serum A-hCG levels during follow-up. However, it rarely arises as a primary entity^6,7^.

In this case, the presence of trophoblastic tissue in the rectum is notable, as no similar cases have been reported in the literature. Unexpected growth of the pregnancy adjacent to the rectum suggests local invasion.

Methotrexate is commonly used as the first-line treatment for this condition because it can effectively disrupt rapidly dividing trophoblastic cells. However, in rare cases, where the patient experiences severe bleeding from the site of implantation, surgical intervention may be necessary^6^.

It is important to note that our case has some limitations owing to the low-resource setting in which it occurred. Laparoscopy is not widely available in Syria because of financial constraints, which has led to the use of laparotomy instead. And the patient did not receive proper antenatal care which led to a delay in diagnosis. Moreover, the absence of a histopathological examination may limit the results of this report.

## Conclusion

This case presents a valuable opportunity to gain a deeper understanding of the etiology of this rare and underrecognized medical condition. It also emphasizes the significance of considering methotrexate as a treatment for persistent trophoblastic tissue as surgery may not be sufficient and closely monitoring serum A-hCG levels, particularly when methotrexate is not contraindicated. Additionally, this report highlights the importance of antenatal care seeing that early detection of the ectopic pregnancy would have provided the option of conservative treatment.

## Ethical approval

Ethical approval was not necessary as our institution waives ethical approval for case reports.

## Consent

Written informed consent was obtained from the patient for publication of this case report and accompanying images. A copy of the written consent is available for review by the Editor-in-Chief of this journal on request.

## Source of funding

No funding from an external source supported the publication of this case report.

## Author contribution

K.Z. and M.N. contributed to drafting the manuscript and undertook a literature review. M.T., R.A., and Y.A.-D. contributed to patient care, conception of the case report, acquisition and interpretation of data, drafting of the manuscript, and critical revision of the article for important intellectual content. B.K. led the surgical procedure and supervised the writing of the manuscript scientifically and academically. All authors approved the final submitted manuscript.

## Conflicts of interest disclosure

The author declares no conflicts of interest.

## Research registration unique identifying number (UIN)

This is a case report; therefore, it did not require a research registration.

## Guarantor

Kareem Zabad and Massa Tarakji.

## Availability of data and materials

All data are included in this article and the online supplementary material. Further inquiries can be directed to the corresponding authors.

## Provenance and peer review

Not commissioned, externally peer-reviewed.
